# Evolution of Interdisciplinary Approaches Among Research-Oriented Universities in Vietnam Toward a Modern Industrial Economy: Exploratory Study

**DOI:** 10.2196/38591

**Published:** 2022-08-17

**Authors:** Bach Xuan Tran, Long Hoang Nguyen, Hao Anh Si Nguyen, Thuc Minh Thi Vu, Anh Linh Do, Lien Thi Khanh Nguyen, Nga Thanh Ngoc Kim, Trang Thu Hong Trinh, Carl Latkin, Cyrus S H Ho, Roger C M Ho

**Affiliations:** 1 Institute for Preventive Medicine and Public Health Hanoi Medical University Hanoi Vietnam; 2 Bloomberg School of Public Health Johns Hopkins University Baltimore, MD United States; 3 VNU University of Medicine and Pharmacy Vietnam National University Hanoi Vietnam; 4 Institute of Health Economics and Technology Hanoi Vietnam; 5 Vietnam National Innovation Center, Ministry of Planning and Investment Portal Hanoi Vietnam; 6 Harvard Extension School Harvard University Cambridge, MA United States; 7 Department of Psychological Medicine Yong Loo Lin School of Medicine National University of Singapore Singapore Singapore; 8 Institute for Health Innovation and Technology (iHealthtech) National University of Singapore Singapore Singapore

**Keywords:** research, performance, productivity, scientometric, Vietnam, Asia, metric, pattern, journal, publication, publishing, output, science, scientific

## Abstract

**Background:**

Vietnam’s 2045 development plan requires thorough reforms in science and technology, which underlines the role of research-oriented universities in generating and transforming knowledge. Understanding the current research performance and productivity in Vietnam is important for exploiting future agendas.

**Objective:**

This study aims to explore the growth patterns and collaborations in the scientific publications of Vietnam.

**Methods:**

Data on documents in the Web of Science Core Collection database were searched and extracted to examine the research performance in Vietnam. Publication growth patterns in both quantity and quality were examined. The evolution of research disciplines and collaboration networks were also analyzed. Trends in the growth in the number of publications, citations, and average citations per publication between 1966 and 2020 were presented. Temporal tendencies of the 10 most productive research areas in each period were illustrated. VOSviewer software was used to analyze the discipline network, country network, and institution networks. The trends and the geographical distribution of the number of publications and citations were analyzed.

**Results:**

A total of 62,752 documents in 8354 different sources from 1966 to 2020 were retrieved. A substantial growth was observed in the Vietnamese scientific output during this period, which was mainly research with international collaboration. Natural sciences such as mathematics, materials science, and physics were the top 3 most productive research fields during 1966-2020 in Vietnam, followed by experimental research fields such as multidisciplinary sciences, plant sciences, public, environmental, and occupational health. In 1966-2020, there was the emergence of multidisciplinary research–oriented universities in both public and private sectors along with a significant increase in the number of interdisciplinary and multidisciplinary publications. Although the scientific quality has improved, these publications are still of mostly medium quality as they are concentrated in middle-ranking journals.

**Conclusions:**

Our study highlights the notable growth in research performance in terms of both quality and quantity in Vietnam from 1966 to 2020. Building multidisciplinary and interdisciplinary research agenda, developing networks of local and international researchers for addressing specific local issues, improving the participation of private sectors, and developing science and technology mechanisms are critical for boosting the research productivity in Vietnam.

## Introduction

Investment in science and technology plays an important role in the growth of every country. The development of science and technology helps to generate new information and knowledge, leading to a change in the production scale, labor productivity, and economic growth, and thereby improving the country’s competitiveness [1-3]. Since the “Doi Moi” renovation (1986) and especially after joining the Association of Southeast Asian Nations (1995), Vietnam has been increasingly paying attention to scientific research and international publications. In 2017, Vietnam spent 0.53% of the gross domestic product on research and development, which was up from 0.19% in 2011 [4]. There have been many initiatives proposed to promote scientific research and innovation activities in Vietnam, including the establishment of national scientific research support funds such as the National Foundation for Science and Technology Development and the National Technological Innovation Fund, Science and Technology Support Units in Ministries and local governing bodies and educational and research institutions, as well as the participation of private enterprises such as the Vingroup Innovation Foundation. These initiatives require publications in international journals or patents as compulsory outcomes for every research grant. In terms of policy, international publications or patents became a mandatory requirement for doctoral students and their supervisors from 2017 [5] and for promoting the national Professor title since 2018 [6]. In addition, Vietnam’s extensive integration process in the global economy has enabled scientists to access financial support from abroad and strengthen cooperation with other high-income countries such as the United States of America, Europe, Japan, Korea, and Australia [7]. These reasons have contributed to the significant growth in the number of publications in international peer-reviewed journals, thereby improving Vietnam’s position on the global scientific map.

However, science, technology, and innovation in Vietnam will confront many challenges in the coming time. A report “Assessment of Science, Technology, and Innovation in Vietnam,” published by the World Bank pointed out the barriers for the development of science and technology in Vietnam, including (1) limited institutional support framework for science and technology, (2) shortage of high-quality knowledge resources, (3) the immaturity and fragmentation in the policy and research implementation, (4) underappreciation of research and development activities in private and state-owned enterprises, and (5) modest contribution of state universities and research institutions [8]. Although the higher education sector accounts for more than half of the human resources for science and technology (50.8%) and two-thirds of science and technology products, the role of universities in creating knowledge through science and technology activities is still limited [8].

In 2020, the government of Vietnam proposed strategic plans to actively participate in the Fourth Industrial Revolution and promote a knowledge-based economy [9]. The Vietnamese leader has also set a goal of developing the country from 2025 to 2030, with a vision extending to 2045, in which universities that are oriented to multidisciplinary research play a key role in the innovation process and improve the labor productivity of the Vietnamese people. This is a strategic transformation direction, which requires a thorough study of the development process of universities, including the science and technology capacity, to come up with long-term investment plans. Indeed, globally, the involvement of research universities is positively correlated with the economic growth of a nation [10]. It is especially true in low-income countries that the research universities can serve as a bridge in connecting domestic and international social resources, promoting interdisciplinary development and linkage between the economic regions of a country [11].

To be able to propose appropriate policies and models to help promote science and technology activities and the development of research-oriented universities, it is necessary to have assessments of the capacity in scientific research and cooperation through quantity, structure, and international publication quality in Vietnam. Evaluating the effectiveness of investment in science and technology (or the productivity of science and technology) often encounters obstacles because the process of scientific and technological research rarely produces specific commercialized products. Given that the essence of the scientific research process is to generate information and knowledge, scientific productivity can be quantified through the quantity and quality of scientific productions such as patents or publications in peer-reviewed journals. Previous research results showed that the number of scientific publications in peer-reviewed journals is an important indicator of a country’s socioeconomic development status [12-14]. Therefore, this paper used a scientometric approach to explore the growth patterns and collaborations in scientific publications in Vietnam.

## Methods

### Database

This study analyzed the data of Vietnamese scholars’ international scientific papers and publications in the Web of Science Core Collection database system. This database consists of more than 21,100 journals, which are indexed in different classification systems such as Science Citation Index Expanded, Social Sciences Citation Index, Arts & Humanities Citation Index, Emerging Sources Citation Index, and Conference Proceedings Citation Index. This database was chosen because the journals in this system are considered to be of high quality, which is important for measuring the outcomes of academic and policy applications [15,16]. Additionally, this system provides comprehensive information of the scientific papers or publications for analysis. The Web of Science system has been used by many governments and higher education research institutions to assess science and technology productivity and research quality [17,18].

### Searching Strategy

We utilized the tag “CU=(Vietnam) OR CU=(Viet Nam)” for searching data of all publications in the Web of Science Core Collection until December 31, 2020 from Vietnam. These data were downloaded and extracted into the Microsoft Excel software. The criteria for selecting the papers and publications in the study included (1) publications (eg, papers, reviews, conference proceedings, abstracts, commentaries, editorials, news, other types of nonjournal publications) in the Web of Science Core Collection database, (2) published in the English language, and (3) having full information of the authors. Papers were excluded if their author information was not available. Each record contained the following information: authors, address/affiliation of each author, title, abstract, journal, number of citations, time of download, scientific category, research area, and funding. For examining collaboration networks, we categorized the papers into 3 groups: (1) papers with a single author; (2) domestic collaboration, if all authors of the paper had affiliations in Vietnam; and (3) international collaboration, if at least one author had an affiliation in a foreign country.

### Data Analyses

We summarized the characteristics of the publications by using descriptive statistics. Trends in the growth of the number of publications, citations, and average citations per publication between 1966 and 2020 were presented. We also divided the study period into 4 intervals: before 1990, from 1990 to 1999, from 2000 to 2014, and from 2015 to 2020. Temporal tendencies of the 10 most productive research areas in each period were also illustrated. The VOSviewer software (Leiden University's Centre for Science and Technology Studies) was used to analyze the discipline network, country network, and institution networks. Microsoft Excel software was utilized to analyze the trend and geographical distribution of the number of publications and citations.

## Results

### Publication Output and Growth Trends

From 1966 to 2020, Vietnamese scientists had published 62,752 documents in 8354 different sources indexed in the Web of Science Core Collection database. Table 1 shows the general characteristics of the publications. The main type of scientific publications was original papers (58,509/62,752, 93.2%), followed by meeting abstracts (2572/62,752, 4.1%) and review papers (1992/62,752, 3.2%). There were 4791 (7.6%) single-authored documents that were written by 2120 authors. The mean numbers of authors per document, coauthors per document, and documents per author were 1.95, 12.90, and 0.51, respectively.

**Figure 1:**
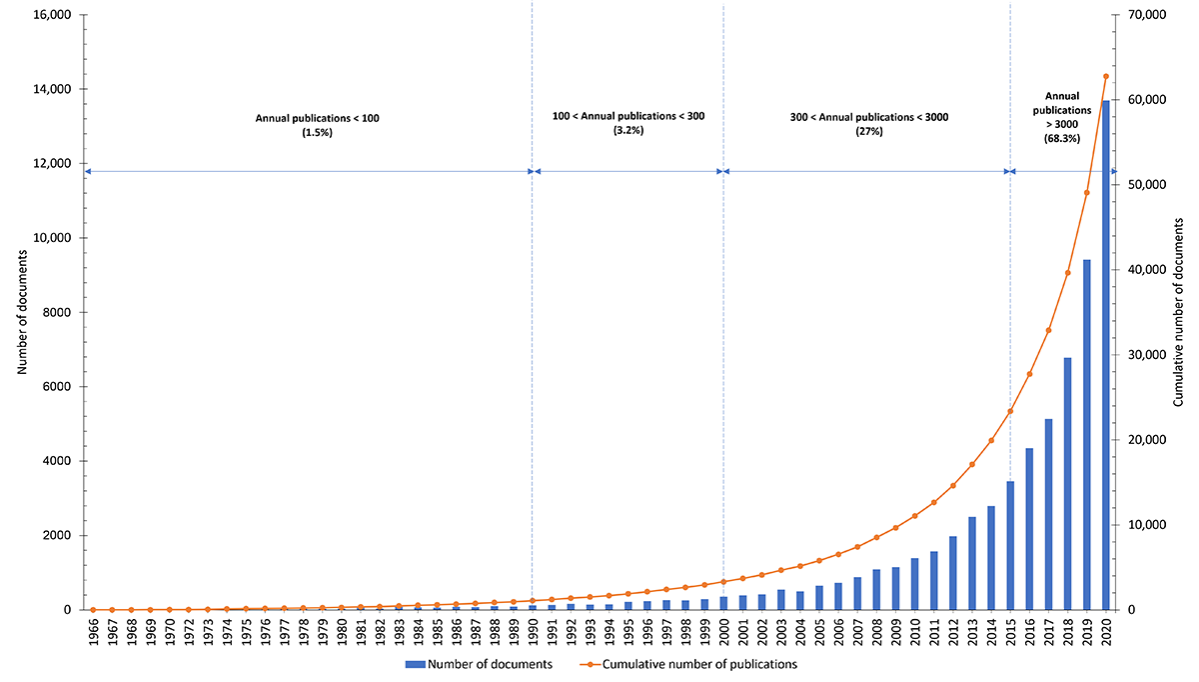
The annual number of publications and the cumulative number of publications in Vietnam.

**Figure 2:**
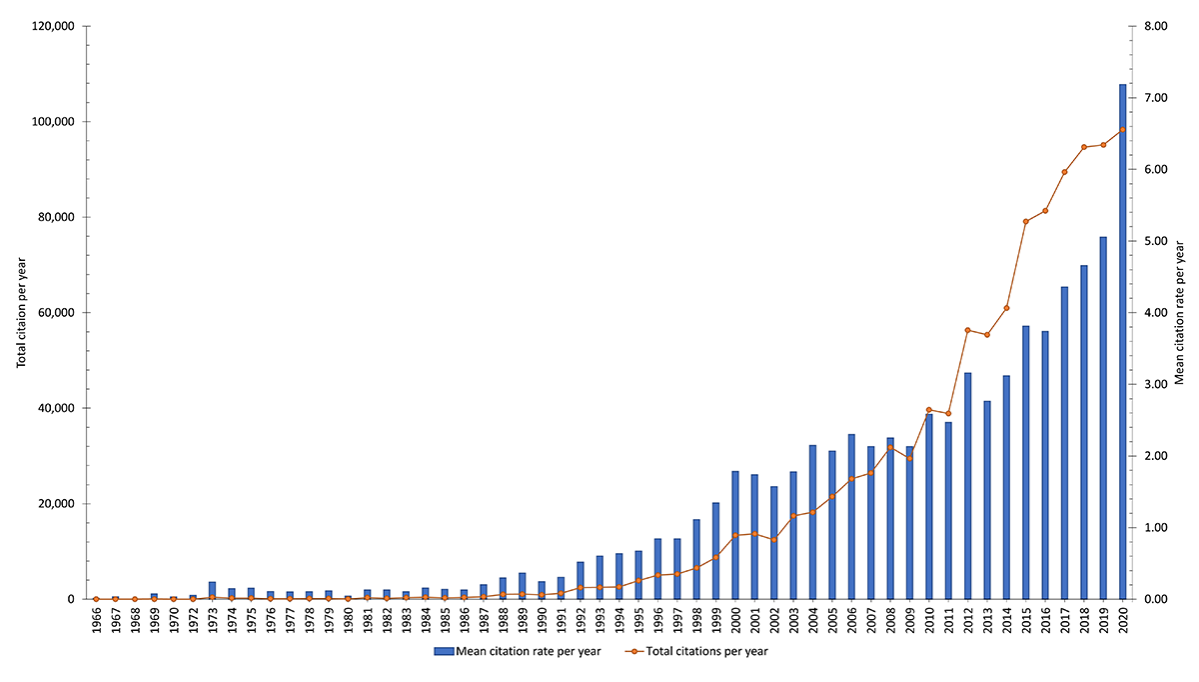
The annual number of citations and mean citation rate per year.

Figure 1 summarizes the growth tendency of the publications in Vietnam. Beginning with 6 documents from 1966, the number of publications grew gradually in the next 2 decades to reach 91 publications in 1989. The number of documents in 1966-1989 accounted for 1.5% (956/62,752) of the total publications from 1966 to 2020. The number of publications increased more rapidly in the next decade (1990-1999) and reached 296 publications in 1999. In the third period from 2000 to 2014, the number of publications remarkably increased compared to that in the second period. By the middle of the third period (2008), the number of publications exceeded 1000 and reached 2793 publications in 2014. The total number of publications in this period accounted for 27% (16,970/62,752) of the total cumulative number of publications. However, in the fourth period (2015-2020), the number of publications exploded and outperformed that in the previous periods, accounting for 68.3% (42,832/62,752) of the total number of publications. Figure 2 shows that there was a substantial increase in the number of citations and the mean citation rate per year. Although publications in 1966 had only 3 citations (for 5 publications) with a mean citation rate at 0.1 per year, in 2020, the total number of citations was 98,362 (for 13,691 publications) and the mean citation rate was 7.18.

**Table 1:** Main information regarding the collection and characteristics of the papers published by Vietnamese scientists.

Description			Value
**Main information (n)**			
	Sources	8354	
	Documents	62,752	
	Mean citations per document	16.64	
	Mean citations per year per document	3.00	
**Document types (N=62,752), n (%)**			
	Original papers	58,509 (93.2)	
	Meeting abstracts	2571 (4.1)	
	Review papers	1992 (3.2)	
	Proceedings papers	1651 (2.6)	
	Other document types	2162 (6.9)	
**Authors (n)**			
	Authors of single-authored documents	2120	
	Authors of multi-authored documents	120,351	
**Number of authors (N=62,752), n (%)**			
	Single-authored documents	4791 (7.6)	
	Multiple-authored documents	57,961 (92.4)	
**Author collaboration (ratios)**			
	Documents per author	0.51	
	Authors per document	1.95	
	Coauthors per document	12.90	
	Collaboration index	2.08	

### Evolution of Research Disciplines

**Figure 3:**
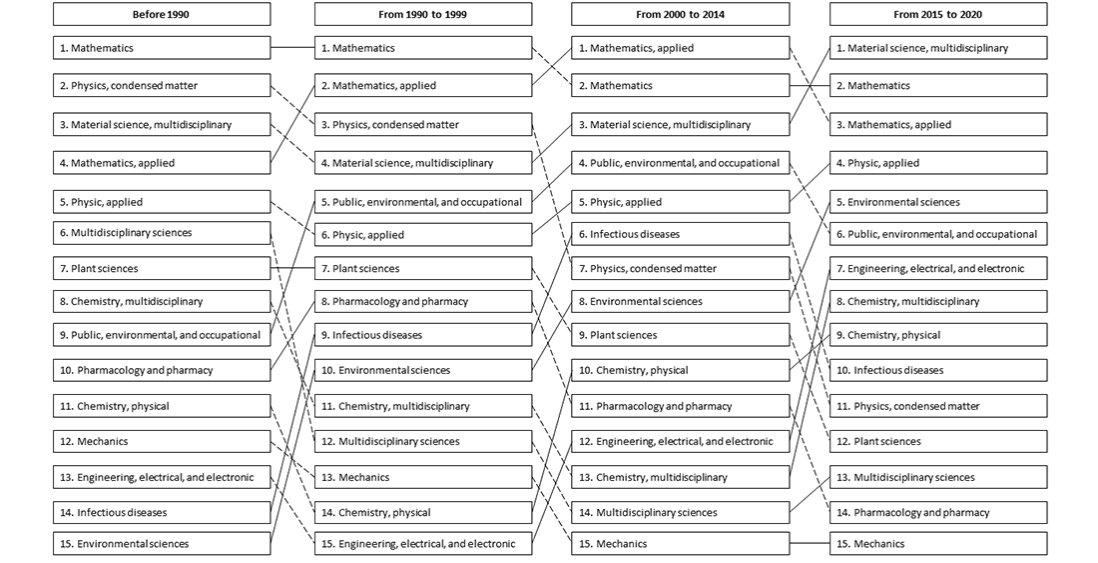
Changes in the top 15 most productive research disciplines in Vietnam. The dotted line indicates that the number of papers in that discipline reduced in the next period, while the solid line indicates that the number of papers in that discipline increased in the next period.

Figure 3 illustrates the shift in the top 15 most productive research disciplines in different periods. Overall, natural sciences such as mathematics, materials science, and physics were the top 3 most productive research fields during 1966 to 2020 in Vietnam, followed by the experimental research fields such as multidisciplinary sciences, plant sciences, public, environmental, and occupational health.

**Figure 4:**
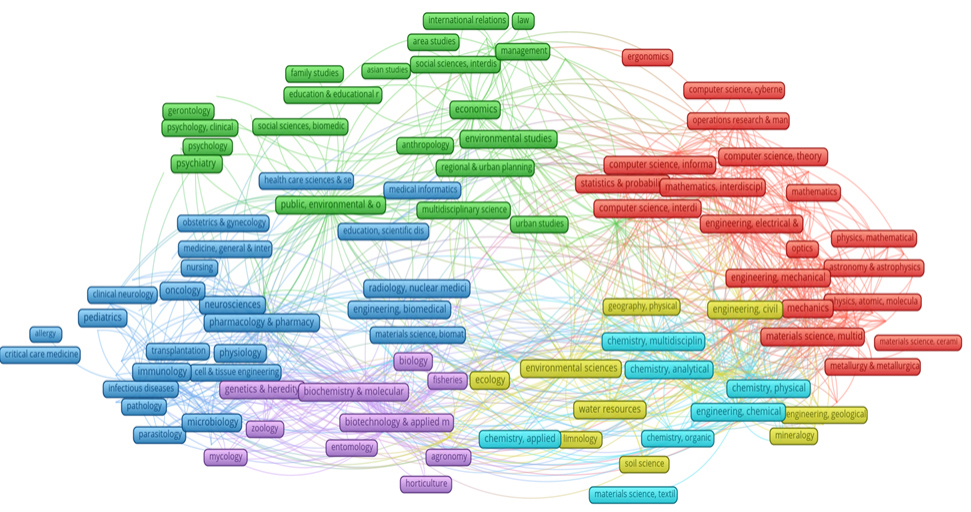
Network of the research disciplines in Vietnam.

Figure 4 shows the network analysis of the research disciplines. Overall, there were 6 main clusters of research fields in the international publications by Vietnam scholars: (1) clinical medicine and biomedicine (dark blue), (2) social sciences (green), (3) bioinformatics and life sciences (purple), (4) mathematics, physics, and materials science (red), (5) chemistry (light blue), and (6) earth science (yellow). Researches were mainly conducted among fields that were relatively similar (such as social sciences–clinical medicine or biomedicine-bioinformatics).

Table 2 shows that in the last 2 periods, multidisciplinary researches had a significantly higher rate of having funding compared to research studies with only 1 discipline. Moreover, the mean citation rate per document among studies with 1 discipline was lower than that in other groups in 2015-2020, but the difference was relatively small.

**Table 2:** Number of documents, documents with funding, and mean citation rate per document according to the number of research disciplines.

Characteristics		Number of research disciplines		
		1 discipline	2-3 disciplines	>3 disciplines
**Documents (n)**				
	Before 1990	650	297	9
	1990-1999	1070	863	61
	2000-2014	8302	8139	529
	2015-2020	22,723	18,260	1849
**Documents with funding, n (%)**				
	Before 1990	0 (0)	0 (0)	0 (0)
	1990-1999	24 (2.2)	45 (5.2)	0 (0)
	2000-2014	4137 (49.8)	3993 (49.1)	289 (54.6)
	2015-2020	14,305 (63)	12,498 (68.4)	1387 (75)
**Mean citation rate per document**				
	Before 1990	5.56	8.01	9.00
	1990-1999	18.82	21.34	10.28
	2000-2014	28.35	26.08	24.87
	2015-2020	12.43	12.69	12.92

### Journals

Table S1 of Multimedia Appendix 1 shows the top 15 most active journals (of the 8354 sources of publications) in publishing researches from Vietnam based on the total number of publications. Overall, most of these journals were middle-ranking journals (second to third quartile according to the Web of Science classification). Consistent with the result above, the number of publications in these journals increased significantly from 2000 to 2020. Notably, in recent years, mega journals such as PLOS One, Scientific Reports, or Sustainability seem to be pervasive with a high number of publications from Vietnam compared to other specialized journals.

### Contribution of Institutions and Localities

Table S2 in Multimedia Appendix 1 presents the top 20 most productive Vietnamese institutions. It should be noted that 1 author could have 2 or more addresses or could be affiliated with 2 or more institutions. Vietnam Academy of Science and Technology was the most productive institution (with 11,080 documents, accounting for 14.38% of the total national publications). Seventeen other institutions had more than 1000 documents published in international sources. Nine institutions were located in the northern region, while 8 institutions were located in the southern region, and 3 institutions were located in the central region of Vietnam. Among 20 institutions, only 2 universities (Ton Duc Thang University and Duy Tan University) were private institutions.

Table 3 depicts the geographical variations in the documents having Vietnamese scholars as the first author or as the corresponding author. In general, Hanoi, Ho Chi Minh City, Da Nang, Thua Thien Hue, Can Tho, and Thai Nguyen were the main cities with the highest number of publications that met this condition, and all of these cities had more than 1000 papers in total. These provinces also had the highest number of publications with Vietnamese scholars as corresponding authors. Notably, almost all provinces had lower mean citation rates among documents with Vietnamese as the first author and corresponding authors than the mean citation rate of 16.64 for all published documents in Vietnam, as shown in Table 3.

**Table 3:** Total number of documents and the mean citations per document in 63 provinces.

Order	Total number of documents			Vietnamese as the corresponding author			Vietnamese as the first author		
	Provinces	Documents (n)	Mean citation per document	Provinces	Documents (n)	Mean citation per document	Provinces	Documents (n)	Mean citation per document
1	Ha Noi	31,245	17.5	Ha Noi	13,929	9.6	Ha Noi	7968	7.5
2	Ho Chi Minh city	23,457	18.7	Ho Chi Minh city	12,995	13.3	Ho Chi Minh city	5527	7.3
3	Da Nang	5792	17.9	Da Nang	2449	13.0	Da Nang	543	6.6
4	Thua Thien Hue	1876	16.9	Thua Thien Hue	686	6.4	Thua Thien Hue	369	5.2
5	Can Tho	1646	16.9	Can Tho	459	7.6	Thai Nguyen	260	4.4
6	Thai Nguyen	1134	7.9	Thai Nguyen	372	6.1	Can Tho	242	5.5
7	Hai Phong	650	10.6	Nghe An	163	5.1	Nghe An	202	4.8
8	Binh Duong	465	10.2	Binh Duong	162	7.8	Thanh Hoa	155	7.3
9	Dong Thap	431	16.1	Binh Dinh	137	6.5	Binh Dinh	132	5.6
10	Khanh Hoa	411	14.9	Dong Thap	128	10.3	Binh Duong	109	4.4
11	Nghe An	400	7.6	Hai Phong	127	7.8	Lam Dong	94	6.2
12	Dong Nai	300	12.0	Bac Giang	103	8.9	Dong Thap	81	6.8
13	Binh Dinh	292	9.0	Khanh Hoa	96	11.8	Dong Nai	67	6.6
14	Thanh Hoa	279	10.4	Lam Dong	85	7.7	Hai Phong	65	2.6
15	Lam Dong	229	11.7	Dong Nai	78	8.7	Thanh Hoa	48	11.8
16	Vinh Phuc	204	7.0	Thanh Hoa	72	10.1	Quang Binh	37	6.5
17	An Giang	198	10.1	An Giang	60	5.5	Hung Yen	30	7.1
18	Hung Yen	194	8.2	Dien Bien	52	7.9	Son La	25	3.0
19	Dak Lak	174	10.7	Dak Lak	49	9.8	Dak Lak	24	9.0
20	Bac Ninh	173	16.3	Quang Binh	48	6.2	Ba Ria-Vung Tau	23	12.0
21	Phu Tho	155	12.2	Ba Ria-Vung Tau	43	13.7	An Giang	21	6.9
22	Quang Binh	138	6.4	Hung Yen	39	7.9	Phu Tho	19	4.5
23	Long An	133	6.9	Tra Vinh	38	4.6	Quang Nam	16	4.9
24	Thai Binh	132	34.2	Vinh Phuc	36	4.1	Tien Giang	14	3.0
25	Tra Vinh	132	6.5	Bac Ninh	35	16.4	Ninh Bình	10	8.3
26	Ba Ria-Vung Tau	128	11.1	Phu Tho	33	5.8	Tuyen Quang	10	3.7
27	Tien Giang	114	18.4	Son La	30	8.8	Phu Yen	8	1.3
28	Son La	113	93.0	Long An	23	6.7	Thai Binh	7	16.4
29	Hai Duong	105	6.8	Thai Binh	20	7.2	Hai Duong	6	0.5
30	Dien Bien	98	7.0	Phu Yen	19	6.5	Long An	6	4.0
31	Quang Tri	79	5.6	Quang Nam	17	5.8	Ninh Thuan	6	2.5
32	Quang Ninh	78	10.1	Hai Duong	15	6.8	Vinh Long	6	0.5
33	Ninh Binh	71	20.4	Tien Giang	14	6.9	Bac Ninh	5	18.4
34	Phu Yen	71	8.1	Tuyen Quang	13	2.4	Ha Tinh	4	0.0
35	Quang Nam	67	7.1	Kon Tum	12	6.8	Quang Ngai	4	1.5
36	Binh Thuan	50	19.2	Quang Tri	12	3.6	Quang Ninh	4	1.0
37	Tuyen Quang	50	5.4	Vinh Long	11	0.9	Quang Tri	4	2.0
38	Vinh Long	50	7.6	Ninh Binh	10	9.9	Vinh Phuc	4	0.3
39	Quang Ngai	46	8.2	Ninh Thuan	8	2.8	Kien Giang	3	1.3
40	Kien Giang	44	11.3	Quang Ngai	7	5.4	Binh Thuan	2	1.5
41	Ninh Thuan	37	19.9	Ha Tinh	6	0.3	Ha Giang	2	2.0
42	Ha Tinh	33	8.6	Hau Giang	5	5.0	Ha Nam	2	6.5
43	Gia Lai	32	4.0	Nam Dinh	4	3.0	Kon Tum	2	24.5
44	Kon Tum	32	12.2	Ha Nam	3	7.3	Lao Cai	2	0.0
45	Nam Dinh	29	5.0	Kien Giang	3	3.3	Nam Dinh	2	0.0
46	Ha Nam	27	14.7	Quang Ninh	3	2.3	Soc Trang	2	20.5
47	Ca Mau	25	17.9	Binh Phuoc	2	2.5	Bac Giang	1	0.0
48	Lao Cai	24	5.6	Binh Thuan	2	8.0	Ben Tre	1	5.0
49	Hoa Binh	23	7.0	Hoa Binh	2	0.5	Ca Mau	1	0.0
50	Hau Giang	21	5.4	Lao Cai	2	0.0	Dien Bien	1	0.0
51	Soc Trang	19	10.7	Soc Trang	2	30.0	Gia Lai	1	0.0
52	Ben Tre	17	9.9	Yen Bai	2	3.0	Hau Giang	1	2.0
53	Bac Giang	16	6.1	Ben Tre	1	3.0	Lang Son	1	0.0
54	Binh Phuoc	16	12.3	Ca Mau	1	6.0	Tay Ninh	1	2.0
55	Lang Son	16	17.1	Gia Lai	1	1.0	Bac Kan	0	N/A^a^
56	Bac Lieu	15	6.3	Ha Giang	1	1.0	Bac Lieu	0	N/A
57	Ha Giang	11	8.5	Lang Son	1	5.0	Binh Phuoc	0	N/A
58	Tay Ninh	6	5.7	Bac Kan	0	N/A	Cao Bang	0	N/A
59	Yen Bai	6	13.7	Bac Lieu	0	N/A	Dak Nong	0	N/A
60	Lai Chau	4	1.3	Cao Bang	0	N/A	Hoa Binh	0	N/A
61	Bac Kan	2	4.0	Dak Nong	0	N/A	Lai Chau	0	N/A
62	Cao Bang	2	19.5	Lai Chau	0	N/A	Tra Vinh	0	N/A
63	Dak Nong	2	7.0	Tay Ninh	0	N/A	Yen Bai	0	N/A

^a^N/A: not applicable.

### International Collaborations

According to the retrieved data set, Vietnam had collaborations with more than 200 countries and territories from 1966 to 2020. Table S3 in Multimedia Appendix 1 shows the top 15 most productive countries, which had collaborations with Vietnamese scholars. Based on the total number of publications (62,752 publication), South Korea ranked first with 3716 documents (5.9%), followed by Japan (3625/62,752, 5.8%), China (3388/62,752, 5.4%), and the United States of America (3049/62,752, 4.9%).

**Figure 5:**
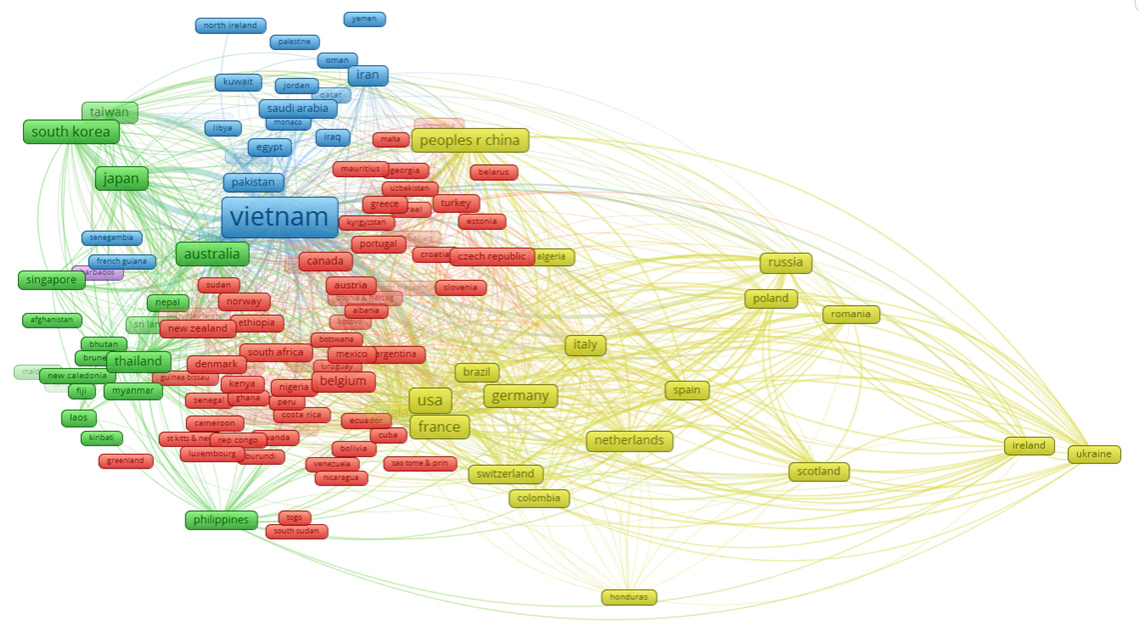
Cooperation network of partner countries and Vietnam.

Figure 5 illustrates the networks of collaborations with Vietnam in research. The size of the box indicates the number of publications, while the thickness of the line shows the strength of the collaborations. The collaborations were divided into 4 clusters. The red cluster indicates strong connections with European (eg, Portugal, Greece, Turkey), African (eg, Ethiopia, Kenya), and South American countries (eg, Venezuela, Cuba). The green cluster is primarily formed by eastern and Southeast Asian countries (eg, South Korea, Japan, Singapore). The blue cluster includes countries from the Middle East (eg, Saudi Arabia, Iraq, Iran). The yellow cluster is formed by other countries (in no particular geographical grouping), including the United States of America, China, or France.

Table 4 shows the trend of the collaborative status in document publishing in Vietnam. In general, the levels of international collaboration in all 3 types, namely, first author, corresponding author, and collaborative status, have decreased significantly over time. The proportion of research with international authors being funded was significantly higher than that of studies with only domestic authors, especially in the period of 2015-2020. Notably, studies with Vietnamese as the first or corresponding authors have a higher degree of influence (through citations) than those with foreign scholars as the first and corresponding authors.

**Table 4:** Number of documents, number of documents with funding, and mean citation rate per document published from 1966 to 2020 in different periods.

Characteristics			First author							Corresponding author							Collaborative status				
			Domestic		International		Relative index^a^		Domestic			International		Relative index		Domestic			International		Relative index
**Documents, n (%)**																					
	Before 1990 (n=965)	4 (0.42)		952 (99.58)		238.00		513 (53.66)			443 (46.34)		0.86		20 (2.09)			936 (97.91)		46.80	
	1990-1999 (n=1994)	108 (5.42)		1886 (94.58)		17.46		749 (37.56)			1245 (62.44)		1.66		150 (7.52)			1844 (92.48)		12.29	
	2000-2014 (n=17,970)	3127 (18.43)		13,843 (81.57)		4.43		6303 (37.14)			10,667 (62.86)		1.69		3341 (19.69)			13,629 (80.31)		4.08	
	2015-2020 (n=42,832)	15,421 (36)		27,411 (64)		1.78		24,596 (57.42)			18,236 (42.58)		0.74		10,861 (25.36)			31,971 (74.64)		2.94	
**Documents with funding, n (%)**																					
	Before 1990	0 (0)		0 (0)		N/A^b^		0 (0)			0 (0)		N/A		0 (0)			0 (0)		N/A	
	1990-1999	6 (5.56)		63 (3.34)		0.60		16 (2.14)			53 (4.26)		1.99		1 (0.67)			68 (3.69)		5.53	
	2000-2014	1739 (55.61)		6680 (48.26)		0.87		3247 (51.52)			5172 (48.49)		0.94		1609 (48.16)			6810 (49.97)		1.04	
	2015-2020	9713 (62.99)		18,477 (67.41)		1.07		15,177 (61.71)			13,013 (71.36)		1.16		6391 (58.84)			21,799 (68.18)		1.16	
**Mean citation rate per document**																					
	Before 1990	2.25		4.10		1.82		3.96			2.39		0.60		5.80			0.55		0.10	
	1990-1999	13.99		5.66		0.40		10.45			9.20		0.88		8.09			11.56		1.43	
	2000-2014	18.82		8.34		0.44		18.45			8.70		0.47		13.33			13.82		1.04	
	2015-2020	8.30		4.26		0.51		9.34			3.22		0.34		5.69			6.87		1.21	

^a^Relative index was calculated by dividing the proportion of documents with international collaboration to the proportion of documents with domestic collaboration or by dividing the mean citation rate per document with international collaboration to the mean citation rate per document with only domestic collaboration.

^b^N/A: not applicable.

## Discussion

### Overview

A knowledge-based economy requires a country to heavily invest in science and technology in addition to receiving technology transferred from other countries. Developing science and technology based on Vietnam’s internal resources is an essential component for promoting economic growth and creating breakthroughs in productivity, quality, efficiency, and increasing competitiveness for sustainable socioeconomic development. This study reviewed the development of research performance in Vietnam in the period from 1966 to 2020, thereby identifying the trends in the development of science and technology in Vietnam, the contribution of research-oriented universities, and the discovering factors that need to be addressed to improve and enhance the science and technology capacity of Vietnam.

### Principal Findings

Our results indicated that Vietnam’s research productivity has been improving significantly, especially in the recent 5 years (from 2015 to 2020). This is reflected in the number of scientific documents in 2020 (13,691 documents), which increased by nearly 50% compared to that in 2019 (9415 documents). This can be explained by the fact that during this period, specific policies and activities aimed at promoting innovation in Vietnam from the government side as well as the formation of science funds have made significant contributions to the development of science and technology. In addition, in some research-oriented universities, policies on money rewards of universities and research institutes for scientific papers in international journals increased the motivation and research performance of Vietnamese researchers. However, despite the significant progress, the knowledge contribution of Vietnamese scholars is still modest compared to those in other countries even in the Association of Southeast Asian Nations region, such as Malaysia (24,423 documents in 2020), Thailand (16,697 documents in 2020), or Singapore (23,225 documents in 2020), according to statistics of the Web of Science database [19]. Furthermore, the number of scientific publications as of December 14, 2021 (12,783 publications) was almost equal to that in 2020, showing that science and technology activities in Vietnam are slowing down [19]. This phenomenon could be attributable to the COVID-19 pandemic; however, it might be a concerning indicator showing that current policies are being saturated. Therefore, a breakthrough in the development policy is necessary for promoting Vietnam’s science and technology and the contribution of research-oriented universities in Vietnam.

With a large population (nearly 100 million people) and large socialization resources from the private sector, Vietnam’s potential for science and technology development is substantial. However, in Vietnam, the proportion of higher education (eg, undergraduate, postgraduate education) accounts for less than 30%, and the rate of the population engaged in research and development activities was only 887 people per million people in 2017, which is much lower than that in Thailand (1632 per million people in 2016) or Malaysia (2859 per million people in 2016) [8]. In addition, the results of this study showed that the role of private research institutions was not strong in the period of 1966-2020. In the top 20 research institutions with the highest scientific productivity, there were only 2 private universities. A report by the Ministry of Science and Technology showed that the innovation level of private organizations in Vietnam was lesser than expected when compared to that in other countries with similar income per capita; for example, only 53% of Vietnamese innovators informed new products in the market, while this proportion was 75% in Malaysia and 86% in Thailand [8]. This can be explained by the fact that the majority of private universities are mainly application-oriented universities, which were suitable for career guidance, while there were only few research-oriented universities [20]. However, currently, many private universities have announced the development of strong research groups with large investments. This is a model that has proven effective in other countries around the world [21-24]. Therefore, the launch of private universities in the science and technology map of Vietnam is expected to be more common in the coming period.

Findings in the analysis of journals and citations depicted those Vietnamese studies that were mainly published in middle-ranking journals. In addition, although research quality had improved with an increase in the mean number of citations per document over the years, this level is still not comparable to that in other countries in the Association of Southeast Asian Nations region [12]. Promoting interdisciplinary research is an effective way to improve research quality [25,26]. In this study, the results showed the shift in the priority research fields of Vietnam over the period of this study. In the period before 2015, research topics focused on theoretical areas such as mathematics or physics. However, in the recent period, research has been performed and published more frequently in the experimental and applied fields. Interdisciplinary and multidisciplinary research has been becoming a trend gradually in response to the increasingly complex domestic and international contexts. The involvement of multidisciplinary fields aids to solve problems from different aspects of issues, thereby significantly improving the quality of research. For example, research related to the topic of COVID-19 in 2020 involved the fields of biomedicine, bioinformatics, and mathematics to build epidemiological models for early warnings [27]. Therefore, policies that promote multidisciplinary and interdisciplinary research should also be included in science and technology policies.

International publications are contributed by major academic centers in major localities such as Hanoi, Ho Chi Minh City, Da Nang, Hue, Thai Nguyen, and Can Tho. This can be explained by the low level of English literacy of scientists in other localities [28]. However, this is mainly due to limited resources being available, and most of the resources are distributed in the provinces where strong research centers are concentrated. This may lead to a lack of science and technology development in other regions, especially in the mountainous and coastal provinces. However, these are the localities with specific scientific research problems that are valuable in contributing to global knowledge. Indeed, scientific evidence on natural and sociocultural issues in these vulnerable localities in the Web of Science Core Collection database is still limited. Therefore, developing key science programs to promote research strengths in these regions should be prioritized in national science and technology agendas. In addition, the cooperation network between strong research centers and local research units should be strongly encouraged to improve the scientific productivity and scientific research capacity of research institutions in these less developed regions.

Similar to those in previous analyses, findings in this study showed that despite a gradual decline in recent years, the contribution of international collaborations to the total number of publications is huge (through the position of the first author, corresponding authors, and fundings) [7,22,23]. This is especially true in empirical research fields, which require great investment in equipment and technology as well as technology transfer activities from countries with more advanced science. In practice, international collaborations are indispensable for promoting science and technology. These partnerships facilitate access to high-quality human, financial, and infrastructure resources for conducting research [29]. This is reflected in that documents with international collaborations had a higher mean citation per document than documents with only domestic collaborations. This result is similar to that reported in some previous studies, which showed that studies with international collaborations received more attention and had more citations [30,31]. Therefore, participating in global scientific and technological networks and calling for the participation of scientists around the world is essential. Noticeably, from 1990 onwards, documents with Vietnamese as the first author and corresponding author had higher mean citations per document in comparison with documents in which international scholars were the first or corresponding authors. This interesting result further confirms that the role of Vietnamese in leading research should be promoted.

### Implications

This study had several implications for developing policies to improve science and technology not only in Vietnam but also in other low-income countries with a similar socioeconomic background. Currently, Vietnam is facing 2 major challenges. In the global context, the post–COVID-19 pandemic economic downturn and the rapid change in science and technology during the Fourth Industrial Revolution have greatly influenced the development of Vietnam owing to the country’s strong participation in the global economy [8]. In the domestic context, the shortage of high-quality human resources, limited financial resources, and inappropriate policy mechanisms are the critical barriers to research and innovation. According to a report by the Ministry of Science and Technology of Vietnam and the World Bank, in the coming years, Vietnam will spend at least 2% of gross domestic product on science and technology activities as well as increase the number of people participating in science and technology activities from 887 people per million people to 1100-1200 people per million people [8]. However, national and regional science and technology development plans need to develop strategic activities. Lessons learned from this study suggest that in Vietnam as well as in other countries with similar background, promoting multidisciplinary research and local research network to address local demands, developing rigorous and transparent mechanisms for science and technology activities to meet the international standards, improving socialization in research, as well as actively participating in the global science and technology network would be the solutions for the development of science and technology in the long run.

### Strengths and Limitations

One of the strengths of this study is the detailed analysis of the characteristics of every single document, regarding authors, journals, citations, and affiliations. Besides, we analyzed the publications in Vietnam for a long period (from 1966 to 2020), which allowed us to understand the patterns of publication growth and collaborations in Vietnam. However, several limitations should be acknowledged. First, our analysis was mainly based on the Web of Science data set with English language documents. Thus, documents from sources that were not indexed in the Web of Science database were not included. Second, given the constrained English knowledge of Vietnamese scholars, our analysis might underestimate the true science and technology output in Vietnam. More rigorous studies with other databases, different languages, and other methods (such as desk review or qualitative interviews) for measuring science and technology should be performed to elucidate the status of science and technology in Vietnam as well as in other countries.

### Conclusion

Our study highlights the substantial growth in the Vietnamese scientific output from 1966 to 2020. The published documents were mainly concentrated in several strong scientific research institutions in a few localities of Vietnam and were often published in middle-ranking journals. International collaborative studies played a major role in improving the quality of research, but Vietnamese-led studies were more influential than other studies led by international authors. Building networks of local and international researchers for addressing specific local issues, promoting the participation of private sectors, and developing science and technology mechanisms are critical for boosting the research productivity in Vietnam.

## Appendix

Multimedia Appendix 1 Supplementary data.
