# Supplementary material for: Evolution of Interdisciplinary Approaches Among Research-Oriented Universities in Vietnam Toward a Modern Industrial Economy: Exploratory Study
Source: Interact J Med Res. 2022 Aug 17;11(2):e38591. doi: 10.2196/38591 (PMC9434394; doi:10.2196/38591)
Supplement: Multimedia Appendix 1 [file ijmr_v11i2e38591_app1.docx]

**Table S1. Top 15 most active journals based on the total number of publications each period from 1966 to 2020**

| **Before 1990** | | | **1990-1999** | | | **2000-2014** | | | **2015-2020** | | |
| --- | --- | --- | --- | --- | --- | --- | --- | --- | --- | --- | --- |
| **No** | **Journal** | **No. of papers** | **No** | **Journal** | **No. of papers** | **No** | **Journal** | **No. of papers** | **No** | **Journal** | **No. of papers** |
| 1 | Physica Status Solidi B-Basic Research | 39 | 1 | Journal Of Magnetism And Magnetic Materials | 41 | 1 | PloS One | 191 | 1 | Scientific Reports | 376 |
| 2 | Physica Status Solidi A-Applied Research | 36 | 2 | Phytochemistry | 40 | 2 | Zootaxa | 158 | 2 | Ieee Access | 374 |
| 3 | Mathematische Nachrichten | 26 | 3 | Transactions Of The Royal Society Of Tropical Medicine And Hygiene | 38 | 3 | Journal Of The Korean Physical Society | 142 | 3 | Journal Of Asian Finance Economics And Business | 327 |
| 4 | Comptes Rendus De L Academie Des Sciences Serie I-Mathematique | 21 | 4 | Journal Of Optimization Theory And Applications | 30 | 4 | Journal Of Magnetism And Magnetic Materials | 139 | 4 | PloS One | 324 |
| 5 | Acta Physica Polonica A | 14 | 5 | Lancet | 28 | 5 | American Journal Of Tropical Medicine And Hygiene | 133 | 5 | Journal Of Electronic Materials | 273 |
| 6 | Doklady Akademii Nauk Sssr | 14 | 6 | Proceedings Of The American Mathematical Society | 28 | 6 | Tropical Medicine & International Health | 115 | 6 | Sustainability | 266 |
| 7 | Biologiya Morya-Marine Biology | 14 | 7 | Physical Review B | 24 | 7 | Journal Of Mathematical Analysis And Applications | 102 | 7 | Applied Sciences-Basel | 259 |
| 8 | Czechoslovak Journal Of Physics | 13 | 8 | Numerical Functional Analysis And Optimization | 20 | 8 | Nonlinear Analysis-Theory Methods & Applications | 99 | 8 | Vietnam Journal Of Chemistry | 219 |
| 9 | Phytochemistry | 12 | 9 | American Journal Of Tropical Medicine And Hygiene | 19 | 9 | Advances In Natural Sciences-Nanoscience And Nanotechnology | 97 | 9 | Rsc Advances | 218 |
| 10 | Journal Of Mathematical Analysis And Applications | 11 | 10 | Nonlinear Analysis-Theory Methods & Applications | 18 | 10 | Journal Of Optimization Theory And Applications | 96 | 10 | Zootaxa | 201 |
| 11 | Anthropological Linguistics | 11 | 11 | Communications In Algebra | 18 | 11 | Physical Review B | 96 | 11 | Energies | 185 |
| 12 | Military Medicine | 11 | 12 | Physica Status Solidi B-Basic Research | 17 | 12 | Aquaculture | 85 | 12 | Science Of The Total Environment | 177 |
| 13 | Revue Roumaine De Mathematiques Pures Et Appliquees | 11 | 13 | Journal Of Algebra | 16 | 13 | Physica B-Condensed Matter | 83 | 13 | International Journal Of Environmental Research And Public Health | 173 |
| 14 | Comptes Rendus Hebdomadaires Des Seances De L Academie Des Sciences Serie A | 10 | 14 | Solid State Communications | 16 | 14 | Journal Of Applied Physics | 81 | 14 | Sensors | 163 |
| 15 | Crystal Research And Technology | 9 | 15 | Journal Of Mathematical Analysis And Applications | 15 | 15 | Journal Of Natural Products | 74 | 15 | American Journal Of Tropical Medicine And Hygiene | 152 |

**Table S2.** Top 20 most productive Vietnamese institutions based on the total number of publications.

| **Order** | **Institutions** | **Location** | **No. of documents** | **%** |
| --- | --- | --- | --- | --- |
| 1 | Vietnam Academy of Science and Technology | North | 11080 | 14.38 |
| 2 | Ton Duc Thang University | South | 9895 | 12.84 |
| 3 | Vietnam National University Ho Chi Minh City | South | 6777 | 8.80 |
| 4 | Duy Tan University | Center | 6751 | 8.76 |
| 5 | Vietnam National University Hanoi | North | 5848 | 7.59 |
| 6 | Hanoi University of Science Technology | North | 3789 | 4.92 |
| 7 | Hue University | Center | 2052 | 2.66 |
| 8 | Hanoi Medical University | North | 1966 | 2.55 |
| 9 | Can Tho University | South | 1914 | 2.48 |
| 10 | Ho Chi Minh City University of Technology | South | 1616 | 2.10 |
| 11 | Nguyen Tat Thanh University | South | 1608 | 2.09 |
| 12 | Ho Chi Minh City University of Medicine and Pharmacy | South | 1575 | 2.04 |
| 13 | Hanoi National University of Education | North | 1532 | 1.99 |
| 14 | University Of Danang | Center | 1359 | 1.76 |
| 15 | Thai Nguyen University | North | 1317 | 1.71 |
| 16 | Vietnam National University of Agriculture | North | 1265 | 1.64 |
| 17 | Industrial University of Ho Chi Minh City | South | 1053 | 1.37 |
| 18 | National Institute of Hygiene and Epidemiology | North | 878 | 1.14 |
| 19 | Ho Chi Minh City University of Economics | South | 853 | 1.11 |
| 20 | Le Quy Don Technical University | North | 802 | 1.04 |

**Table S3.** Top 15 most productive countries

| **Order** | **Country** | **Number of documents** | **% Documents** | **Number of citations** | **Mean citation per document** |
| --- | --- | --- | --- | --- | --- |
| 1 | South Korea | 3716 | 5.9% | 52234 | 14.06 |
| 2 | Japan | 3625 | 5.8% | 60485 | 16.69 |
| 3 | People Republic of China | 3388 | 5.4% | 60425 | 17.84 |
| 4 | Usa | 3049 | 4.9% | 142672 | 46.79 |
| 5 | Australia | 2496 | 4.0% | 44716 | 17.92 |
| 6 | France | 2449 | 3.9% | 58311 | 23.81 |
| 7 | Germany | 1501 | 2.4% | 29211 | 19.46 |
| 8 | United Kingdom | 1140 | 1.8% | 56401 | 49.47 |
| 9 | Belgium | 993 | 1.6% | 20045 | 20.19 |
| 10 | Russia | 934 | 1.5% | 6893 | 7.38 |
| 11 | Netherlands | 858 | 1.4% | 27971 | 32.60 |
| 12 | Iran | 689 | 1.1% | 11848 | 17.20 |
| 13 | Thailand | 668 | 1.1% | 17431 | 26.09 |
| 14 | Sweden | 525 | 0.8% | 12190 | 23.22 |
| 15 | India | 499 | 0.8% | 9371 | 18.78 |
